# Exploring TNFi drug-levels and anti-drug antibodies during tapering among patients with inflammatory arthritis: secondary analyses from the randomised BIODOPT trial

**DOI:** 10.1007/s00296-024-05665-7

**Published:** 2024-07-24

**Authors:** Line Uhrenholt, Mads E. R. Sørensen, Karen B. Lauridsen, Kirsten Duch, Lene Dreyer, Robin Christensen, Ellen-Margrethe Hauge, Anne Gitte Loft, Mads N. B. Rasch, Hans Christian Horn, Peter C. Taylor, Kaspar R. Nielsen, Salome Kristensen

**Affiliations:** 1 Center of Rheumatic Research Aalborg (CERRA), Department of Rheumatology, Aalborg University Hospital, Aalborg, Denmark; 2 Department of Clinical Medicine, Aalborg University, Aalborg, Denmark; 3 Section for Biostatistics and Evidence-Based Research, The Parker Institute, Bispebjerg and Frederiksberg Hospital, Copenhagen, Denmark; 4 Department of Clinical Immunology, Aalborg University Hospital, Aalborg, Denmark; 5 Research Data and Biostatistics, Aalborg University Hospital, Aalborg, Denmark; 6 Research Unit of Rheumatology, Department of Clinical Research, University of Southern Denmark, Odense University Hospital, Odense, Denmark; 7 Department of Rheumatology, Aarhus University Hospital, Aarhus, Denmark; 8 Department of Clinical Medicine, Aarhus University, Aarhus, Denmark; 9 Department of Rheumatology, Odense University Hospital, Odense, Denmark; 10 Nuffield Department of Orthopaedics, Rheumatology and Musculoskeletal Sciences, University of Oxford, Oxford, UK

**Keywords:** Rheumatoid arthritis, Axial spondyloarthritis, Psoriatic arthritis, Tumour necrosis factor inhibitors, Drug tapering, Clinical trial

## Abstract

To evaluate tumour necrosis factor inhibitor (TNFi) drug-levels and presence of anti-drug antibodies (ADAb) in patients with inflammatory arthritis who taper TNFi compared to TNFi continuation. Patients with rheumatoid arthritis, psoriatic arthritis, or axial spondyloarthritis on stable TNFi dose and in low disease activity ≥ 12 months were randomised (2:1) to disease activity-guided tapering or control. Blood samples at baseline, 12- and 18-months were evaluated for TNFi drug-levels and ADAb. In total, 129 patients were randomised to tapering (n = 88) or control (n = 41). Between baseline and month 18, a significant shift in TNFi drug-levels were observed in the tapering group resulting in fewer patients with high drug-levels (change: − 14% [95% CI − 27 to − 1%]) and more with low drug-levels (change: 18% [95% CI 5–31%]). Disease activity was equivalent between groups at 18 months, mean difference: RA − 0.06 (95% CI − 0.44 to 0.33), PsA 0.03 (95% CI − 0.36 to 0.42), and axSpA 0.16 (− 0.17 to 0.49), equivalence margins ± 0.5 disease activity points. ADAb were detected in eight patients, all from the tapering group. TNFi drug-level category or ADAb were not predictive for achieving successful tapering at 18 months. TNFi drug-levels decreased during tapering which indicate adherence to the tapering algorithm. Despite the difference in TNFi drug-levels at 18 months, disease activity remained equivalent, and only few tapering patients had detectable ADAb. These data do not support using TNFi drug-level and/or ADAb to guide the tapering decision but future research with larger trials is needed.

Trial registration: EudraCT: 2017-001970-41, December 21, 2017.

**Supplementary Information:**

The online version contains supplementary material available at 10.1007/s00296-024-05665-7.

## Introduction

In recent years, tapering of tumour necrosis factor inhibitors (TNFi) in patients with inflammatory arthritis (IA) (i.e., rheumatoid arthritis [RA], psoriatic arthritis [PsA], and axial spondyloarthritis [axSpA]) in sustained remission or low disease activity (LDA) have proven effective in reducing the TNFi dose while maintaining acceptable disease activity [1–10]. Even though tapering comes with the risk of flare, acceptable disease activity is regained for the majority after TNFi dose escalation; thus, limiting the risk of persistent flare [3].

The BIODOPT trial recently evaluated disease activity-guided tapering of biologics compared to continuation of biologics as usual care in patients with RA, PsA, or axSpA in sustained LDA. The study demonstrated that one-third of the tapering group could achieve ≥ 50% biological dose reduction without losing disease control [10].

Previous studies have reported higher TNFi drug-levels in patients with RA, PsA, or axSpA to be associated with an improved treatment response [11–16]. Moreover, presence of anti-drug antibodies (ADAb) were associated with lower TNFi drug-levels and lack of efficacy [11–14, 16]. Thus, presence of ADAb is hypothesised to lead to lower TNFi drug-levels due to neutralising of TNFi or increased TNFi clearance; thereby, resulting in less TNFi efficacy. However, the current knowledge on TNFi drug-levels and presence of ADAb in patients with RA, PsA, or axSpA who taper their biological therapy is extremely limited. It has been speculated that lower TNFi drug-levels due to tapering potentially could lead to an increased risk of ADAb development. Thus, disease activity-guided tapering with prolongation of the dosing interval until flare or withdrawal would have a higher risk of ADAb development compared to a fixed one-step, e.g. 25%, tapering. Development of ADAb due to tapering could thereby lead to increased TNFi neutralisation or TNFi clearance which then could induce loss of effectiveness. The only evidence on the subject is a prospective observational study by Chen et al. who reported lower adalimumab drug-levels at 24 weeks after dose-halving in 64 patients with RA and a low frequency of ADAb development (5% [3/64]) [17].

This study aims to evaluate TNFi drug-levels and presence of ADAb in patients with RA, PsA and axSpA who tapered their TNFi treatment using a disease activity-guided algorithm compared with TNFi continuation as usual care in the BIODOPT trial. The primary objective was to compare TNFi drug-level categories at 18 months, the secondary objective was to assess presence of ADAb at 18 months, tertiary objectives were to evaluate TNFi drug-levels and ADAb at 12 months, and exploratory objectives were to identify possible baseline predictors for successful TNFi tapering based on data from the tapering group.

## Methods

### Study design and participants

The BIODOPT trial has previously been reported in details [10, 18]. It was a pragmatic, multicentre, randomised, open-label, equivalence trial of 18 months duration conducted in Denmark. Patients ≥ 18 years old, diagnosed with RA, PsA, or axSpA, on stable biologic dose, and in LDA ≥ 12 months were randomised (2:1) to tapering or control. A sustained, tapered (lower than standard) TNFi dose at enrolment were allowed if the lower dose was kept ≥ 12 months prior to inclusion. The tapering group followed a disease activity-guided algorithm which increased the TNFi dosing interval with approximately 25% every 4 months until flare or withdrawal [10, 18]. However, due to the long dosing interval, infliximab was spaced with two weeks at each infusion. The control group maintained their baseline biological dosing interval but, as usual practise, a small increase was allowed if requested by the patient.

In this secondary analysis reporting, blood samples collected in connection to the baseline, 12- and 18-months visit were analysed. These specific time points were chosen as patients potentially could taper their TNFi to discontinuation after 12 months; thus, TNFi drug-levels were expected to be lowest at the end of the study which could lead to an increased formation of ADAb.

The blood samples were stored in the Danish Rheumatology Biobank. TNFi drug-levels (adalimumab, certolizumab-pegol, etanercept, golimumab, and infliximab) and ADAb were measured by IDKmonitor enzyme-linked immunoassorbant assays, Immundiagnostik AG, Bensheim, Germany. In accordance with the manufacturer’s recommendation, ADAb were considered positive if values were ≥ 10 arbitrary units/mL. The timing of blood sampling was not fixed to the timing of last TNFi administration; however, the date of last TNFi administration was noted at each visit. Only patients treated with a TNFi at baseline were included in these analysis as assays for measuring abatacept or tocilizumab drug-levels not were available.

At 18-months, patients were considered to have successfully tapered their TNFi if the dose was reduced by ≥ 50% compared to baseline, no protocol violations had occurred, and they were in LDA, defined as RA or PsA: Disease Activity Score28-C-Reactive Protein (DAS28-CRP) ≤ 3.2, or axSpA: Ankylosing Spondylitis Disease Activity Score (ASDAS) < 2.1.

### Statistical analysis

These secondary analyses were conducted and reported in accordance with the pre-specified SAP (provided as a supplementary), the CONSORT statement [19, 20] and the TRIPOD statement [21, 22]. The analyses were based on intention-to-treat (ITT) i.e., all randomised participants independent of subsequent protocol deviations.

Baseline characteristics were summarised by count and percentage, mean and standard deviation, or median ad interquartile range according to distribution.

The primary outcome ‘TNFi drug-level’ was evaluated as categorised as very low and very high values were truncated. Based on previous literature [15, 23, 24] or the manufacturer’s recommendation, the variable was divided into ‘low’, ‘intermediate’, and ‘high’, Supplementary Table S1.

Binary outcomes (TNFi drug-levels and ADAb) were analysed using mixed Poisson regression with robust variance estimator with the fixed effects: group (tapering *vs* control), diagnosis, biologic failure history (on biologic number ≤ 2, *or* ≥ 3), centre, time-point (0, 12, or 18 months) and the interaction between group and time. Patient id number were included as random intercept. Continuous outcomes (disease activity) were analysed using a t-test with unequal variance (if normally distributed). An equivalence margin of ± 0.5 disease activity points was pre-specified.

In the primary analysis, missing values for binary outcomes were handled by ‘single-step imputation’; thus, ‘TNFi drug-level category’ was imputed as ‘*intermediate TNFi drug-level’* as this represent the ‘normal range’ for most patients, and’ADAb’ was imputed as ‘*not having developed ADAb’.* To analyse the potential implication of missing data, a sensitivity analysis was conducted where missing values of ‘ADAb’ was handled as ‘*having developed ADAb’*, and missing values of ‘TNFi drug-level category’ as having ‘*low TNFi drug-level’*. The continuous variable ‘disease activity’ were evaluated as observed i.e., missing values were not imputed.

Post-hoc analyses on the primary and secondary outcomes were performed to capture changes within the trial groups from baseline to month 18 (or month 12). Binary outcomes were analysed by McNemar’s test, and continuous outcomes were evaluated by a t-test with unequal variance (if normally distributed). Moreover, a sensitivity analysis on the primary outcome (TNFi drug-level category) was performed to explore potential implications of blood sampling time in relation to the last dose of TNFi.

In the prediction analysis, missing values for ‘successful TNFi tapering’ were imputed as trial failure i.e., *successful tapering was not achieved*. The following baseline variables were included in analysis: female sex, age, Body Mass Index (BMI), diagnosis, disease duration, on conventional synthetic disease-modifying anti-rheumatic drugs (csDMARDs), on ≥ 2 csDMARDs, on methotrexate, repeated biologics failure (on biological agent number ≥ 3), duration of baseline biological therapy, previous biologic tapering attempt, C-reactive protein, in remission (RA and PsA: DAS28-CRP < 2.6, or axSpA: ASDAS < 1.3), TNFi drug-level category, and presence of ADAb. Continuous variables were grouped to identify relevant non-linearity in which case the variable would be categorised into clinically relevant groups by expert opinion. The potential baseline predictors were analysed using univariable modified Poisson regression with robust variance estimator. Variables with a univariate p-value < 0.10 were included in a multivariable, data-driven regression analysis. Moreover, a multivariable, clinical-driven regression analysis including baseline variables judged to be of particular interest by expert opinion (BMI, TNFi drug-level category, presence of ADAb, and on csDMARDs) were also performed. Pairwise correlation between predictors were explored using treelet transformation. Leave-one-out cross-validation was performed to receive the area under the receiving operator curve.

All analyses were performed using commercially available statistical software (STATA, version 18, or SAS, version 9.4).

## Results

**Fig. 1:**
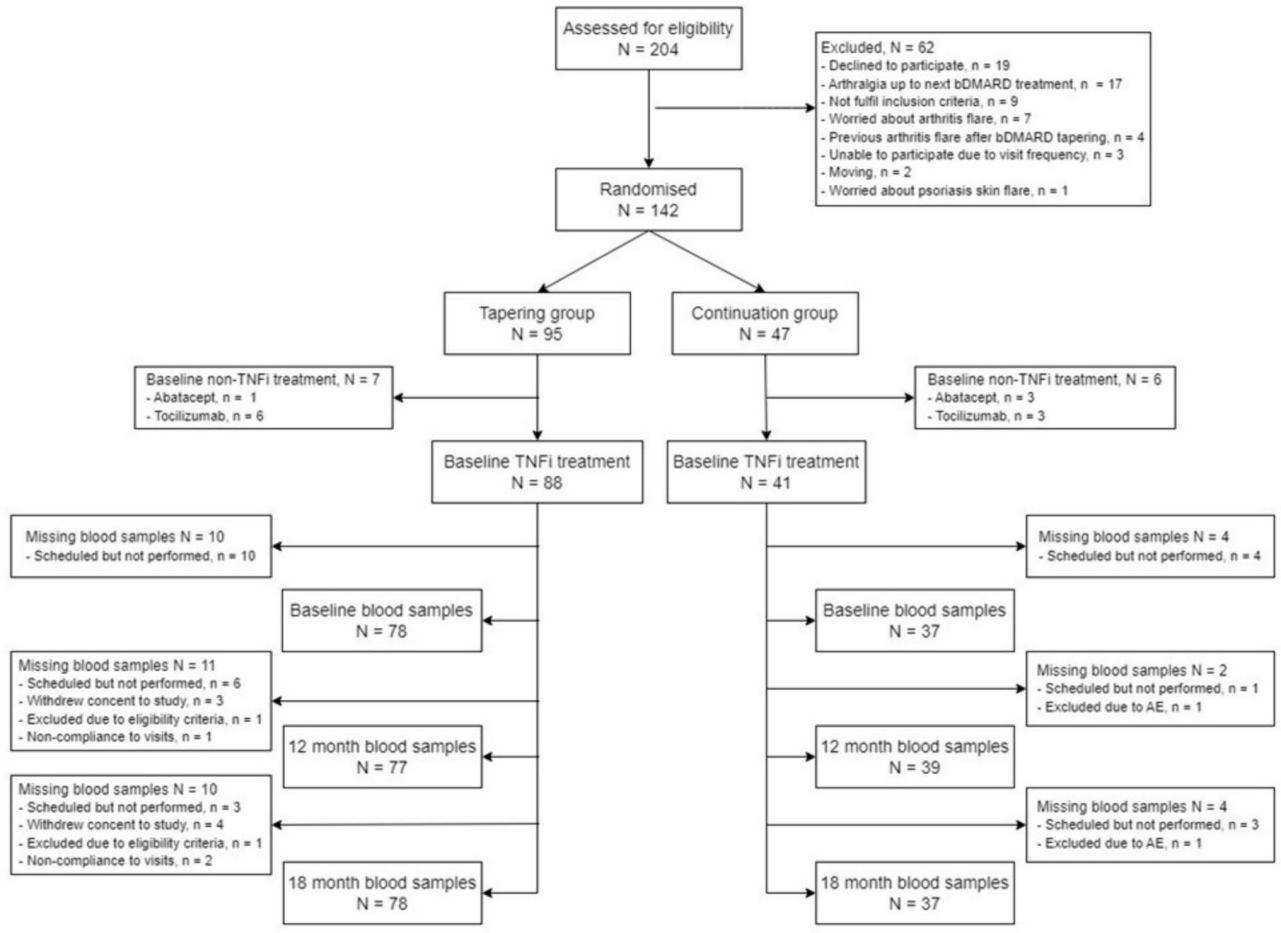
Flow-diagram over blood samples collected during the study period

In total, 129 patients were included in these secondary analyses of which 88 were allocated to the tapering group and 41 to the control group, Fig. 1. Blood samples from 14 patients were missing from baseline despite being scheduled whereas the majority of missing blood samples at 12 and 18 months were due to loss to follow-up e.g., withdrawal of consent to participate, or trial visit not performed due to non-compliance.

As presented in Table 1, baseline characteristics were well-balanced. Notably, the percentage of women in the tapering group was a little higher than in the control group (52% vs 37%). Median BMI was in the overweight range for both groups; median BMI: 25.3 kg/m^2^ vs 26.6 kg/m^2^. A previous TNFi tapering attempt had been done in 30% of the tapering group and 27% of the control group. At inclusion, 19% of the tapering group and 20% of the control group were treated with a sustained, tapered (lower than standard) TNFi dose. Of these patients, 44% (11/25) were diagnosed with RA, 40% (10/25) with axSpA, and 16% (4/25) with PsA. The majority received tapered adalimumab (40% [10/25]) or tapered infliximab (40% [10/25]) whereas 16% (4/25) received tapered etanercept, and 4% (1/25) tapered certolizumab-pegol.

**Table 1:** Baseline characteristics analysed as observed

Variable	Tapering group (*N* = 88)	Control group (*N* = 41)
General characteristics		
Female, n (%)	46 (52%)	15 (37%)
Age (years), mean (SD)	50.7 (14.9)	50.1 (15.7)
Body mass index (kg/m^2^), median (IQR)	25.3 (23.2;29.2)	26.6 (23.3;29.5)
Arthritis characteristics		
Diagnosis		
Rheumatoid arthritis, n (%)	34 (39%)	14 (34%)
Psoriatic arthritis, n (%)	18 (20%)	8 (20%)
Axial spondyloarthritis, n (%)	36 (41%)	19 (46%)
Disease duration (years), median (IQR)	10.9 (5.6;18.0)	12.0 (6.3;19.8)
On csDMARD, n (%)	39 (44%)	18 (44%)
On MTX, n (%)	38 (43%)	16 (39%)
On ≥ 2 csDMARDs, n (%)	2 (2%)	1 (2%)
Repeated biologics failure^a^, n (%)	3 (3%)	1 (2%)
Previous attempt to taper TNFi, n (%)	26 (30%)	11 (27%)
TNFi dose		
Standard TNFi dose, n (%)	71 (81%)	33 (80%)
Tapered TNFi dose, n (%)	17 (19%)	8 (20%)
Duration of baseline biologic (years), median (IQR)	4.5 (2.3;8.5)	5.8 (2.5;11.3)
CRP (mg/L), median (IQR)	3.1 (0.9;3.9)	2.7 (0.6;3.9)
Disease activity		
Rheumatoid arthritis^b^, mean (SD)	1.7 (0.4)	1.8 (0.5)
Psoriatic arthritis^b^, mean (SD)	1.7 (0.3)	1.6 (0.5)
Axial spondyloarthritis^c^, mean (SD)	1.1 (0.4)	1.2 (0.3)
In remission^d^, n (%)	75 (85%)	34 (83%)

Laboratory assessments	(N = 78)	(N = 37)
TNFi drug-level category		
High, n (%)	31 (40%)	16 (43%)
Intermediate, n (%)	19 (24%)	11 (30%)
Low, n (%)	28 (36%)	10 (27%)
Presence of ADAb, n (%)	3 (4%)	0 (0%)

*N*: number, *SD*: standard deviation, *kg*: kilogram, *m*^*2*^: square meters, *IQR*: interquartile range, *csDMARD*: conventional synthetic disease-modifying anti-rheumatic drugs, *MTX*: methotrexate, *TNFi*: tumour necrosis factor inhibitor, *CRP*: C-reactive protein, *mg*: milligram, *L*: liter, *ADAb*: anti-drug antibodies

^a^Patients on biological agent number ≥ 3

^b^Evaluated by Disease Activity Score (DAS)28-CRP

^c^Evaluated by Ankylosing Spondylitis Disease Activity Score (ASDAS)

^d^Evaluated as DAS28-CRP < 2.6 for RA and PsA and ASDAS < 1.3 for axSpA

TNFi drug-level categories were similar between the trial groups at baseline. ADAb were detected in three patients from the tapering group; all had low TNFi drug-levels and were diagnosed with axSpA. Two of these patients were treated with standard dose infliximab and one with standard dose adalimumab. ADAb were not detected in patients from the control group.

### TNFi drug-levels

At 18 months, 22% in the tapering group had a high TNFi drug-level compared to 42% in the control group; the difference was statistically significant, relative risk (RR) 0.53 (95% confidence interval [95% CI] 0.31–0.90), Table 2. No statistically significant between-group difference was observed for intermediate or low TNFi drug-levels. When stratifying by diagnosis, no significant differences in drug-level categories were observed (data not shown).

**Table 2:** Drug-levels and anti-drug antibodies at 18 months

Variable	Tapering group, *N* = *88*	Control group, *N* = *41*	Between group difference
	*N* (%)	*N* (%)	RR (95% CI )
TNFi drug-level category			
High^a^	19 (22%)	17 (42%)	0.53 (0.31–0.90)
Intermediate^b^	25 (28%)	10 (24%)	1.12 (0.60–2.09)
Low^c^	44 (50%)	14 (34%)	1.47 (0.94–2.32)
Presence of ADAb	4 (5%)	0 (0%)	–

	Mean (SD)	Mean (SD)	Mean difference (95% CI)
Disease activity			
Rheumatoid arthritis^d^	1.94 (0.72)	1.99 (0.51)^e^	− 0.06 (− 0.44 to 0.33)
Psoriatic arthritis^d^	1.61 (0.55)^f^	1.58 (0.32)	0.03 (− 0.36 to 0.42)
Axial spondyloarthritig^e^	1.46 (0.70)^h^	1.30 (0.45)^i^	0.16 (− 0.17 to 0.49)

*N* number, *95% CI* 95% confidence interval, *TNFi* tumour-necrosis factor inhibitor, *ADAb* anti-drug antibodies, *SD* standard deviation

^a^Analysed as ‘high’ or ‘not high’ i.e., ‘not high’ equals intermediate AND low TNFi drug-levels

^b^Analysed as ‘intermediate’ or ‘not intermediate’ i.e., ‘not intermediate’ equals low AND high TNFi drug-levels

^c^Analysed as ‘low’ or ‘not low’ i.e., ‘not low’ equals intermediate AND high TNFi drug-levels

^d^Evaluated by Disease Activity Score (DAS)28-CRP

^e^Missing values = 1 (excluded after baseline visit due to an AE)

^f^Missing values = 4 (two patients withdrew consent, one was excluded after baseline due to conflicts with the eligibility criteria, and one had non-compliance to the scheduled visits)

^g^Evaluated by Ankylosing Spondylitis Disease Activity Score (ASDAS)

^h^Missing value = 3 (two patients withdrew consent, and one had non-compliance to the scheduled visits)

^i^Missing value = 2 (two patients did not answer the patient-reported outcomes and therefore the disease activity score could not be calculated)

A sensitivity analysis, carried out to assess potential implication of missing data, showed similar results as the primary analysis, Supplementary Table S2. Another sensitivity analysis, performed to assess the potential implication of the time span between the last biological dose and blood sampling, did not alter the conclusions (data not shown).

**Fig. 2:**
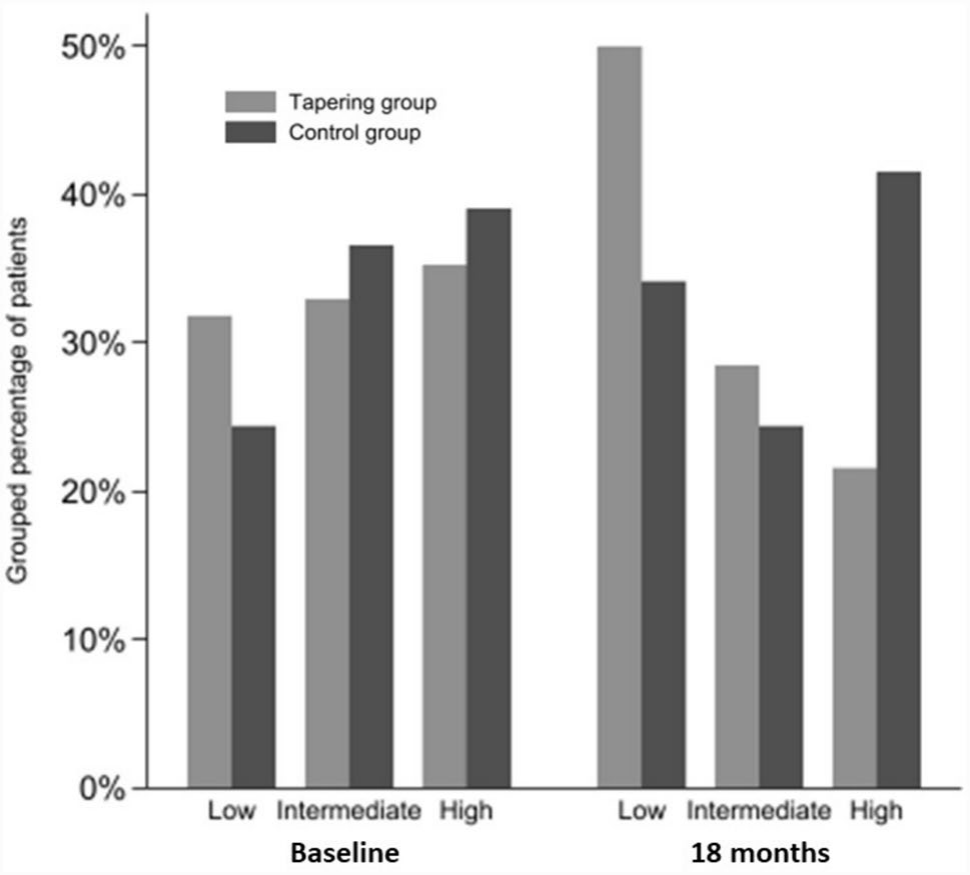
TNFi drug-level categories (low, intermediate, or high) at baseline and 18 months

As presented in Fig. 2, a shift in TNFi drug-levels was observed in the tapering group from baseline to month 18. Thus, fewer patients at 18 months compared to baseline had high TNFi drug-levels (proportional difference: -14% [95% CI − 27 to − 1%]), and more had low TNFi drug-levels (proportional difference: 18% [95% CI 5–31%]), Supplementary Table 3. No significant changes in TNFi drug-levels between baseline and 18 months were noticed in the control group. Thus, these results indicate acceptable compliance to the tapering algorithm.

### Disease activity

Disease activity at 18 months stratified by diagnosis were equivalent as the lower and upper limit of the 95% confidence interval of the between-group difference was within the pre-specified equivalence margin of ± 0.5 disease activity points, Table 2. Thus, a deterioration in disease activity was not observed at 18 months despite the between-group difference in TNFi drug-levels.

### Anti-drug antibodies

**Fig. 3:**
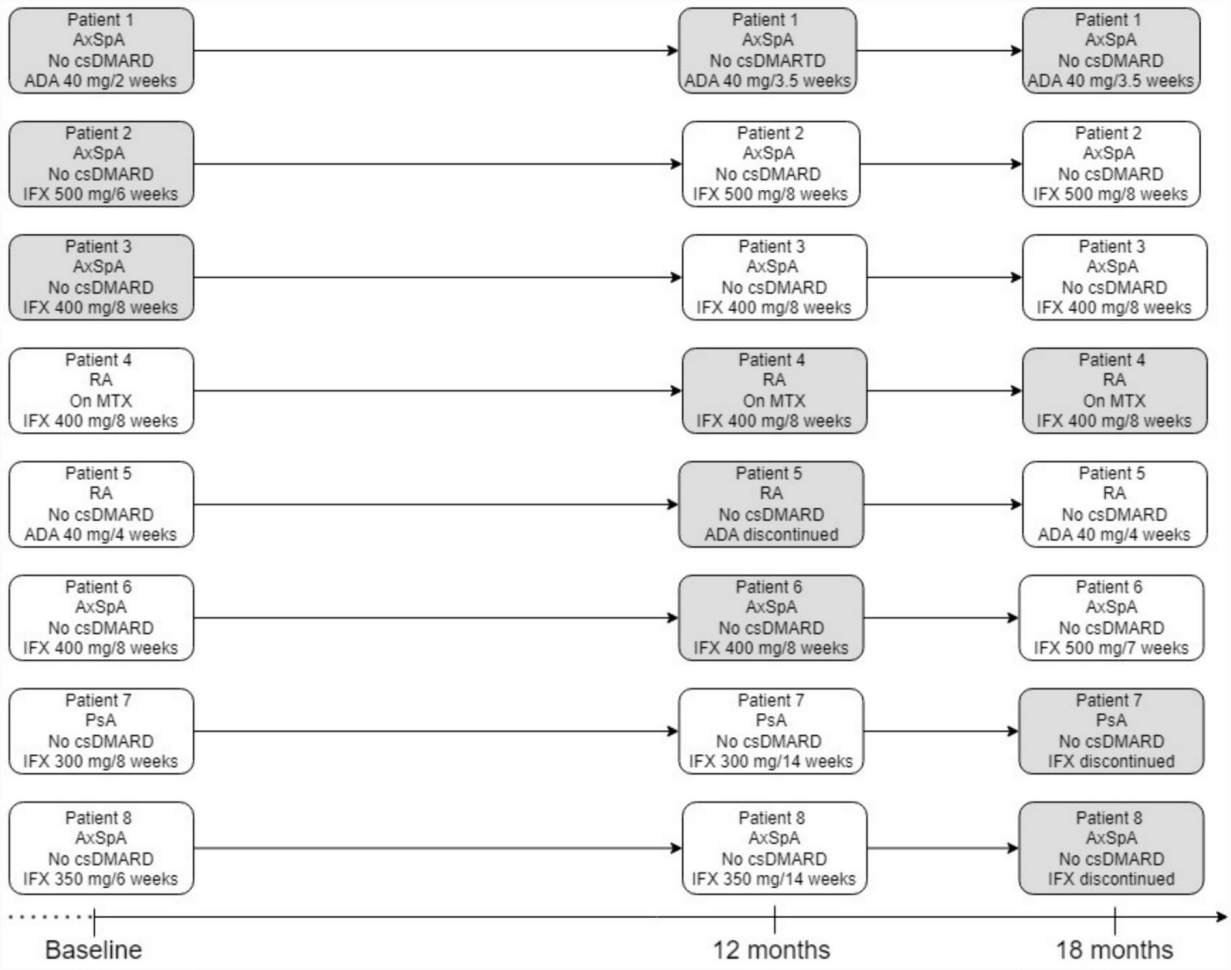
Overview of patients with ADAb during the study period. Presence of ADAb is marked by a grey box

ADAb were detected in eight patients during the study period, all from the tapering group. As presented in Fig. 3, five patients with axSpA (5/36 [14%]), two patients with RA (2/34 [6%]), and one patient with PsA (1/18 [6%]) had detectable ADAb. Patient four was the only one with presence of ADAb who received a csDMARD. All patients with ADAb had low TNFi drug-levels at the time point where ADAb were detected.

Only patient one had ADAb at baseline, month 12, and month 18; the patient managed to taper adalimumab to 40 mg every 3.5 weeks and maintain LDA. Patients two and three had ADAb present at baseline and flared after infliximab was tapered but regained LDA after infliximab was escalated to a tapered dose in patient two and to standard dose in patient three.

Patient four attempted to taper infliximab but flare and developed ADAb afterwards; however, the patient regained LDA after infliximab was escalated back to standard dose.

Patient five tapered adalimumab to discontinuation at month 12 but flared and presented with ADAb; adalimumab was escalated back to baseline dose and LDA regained.

Patients seven and eight tapered infliximab to discontinuation and maintained LDA at month 18 despite detectable ADAb; thus, achieving successful tapering.

Patient six was the only one with detectable ADAb during the study period who was not in LDA at month 18. The patient attempted to taper infliximab but had persistent flared despite escalation back to standard dose; therefore, infliximab was escalated further but the patient did not regain LDA.

### Successful tapering

Successful tapering at 18 months i.e., biological dose reduction by ≥ 50% compared to baseline, no protocol violations, and maintained LDA, were achieved by 32% (28/88) in the tapering group and none in the control group. Post-hoc analyses on the tapering group stratified by diagnosis did not reveal any statistically significant difference in baseline disease activity between patients achieving successful vs non-successful tapering (data not shown).

### Prediction analysis

The prediction analysis was post-hoc limited to the tapering group as no patients in the control group achieved successful tapering. The binomial variables ‘ADAb’ and ‘repeated biologics failure’ could not be included in the analysis due to no events in one group.

In the univariable modified Poisson regression analyses, none of the included baseline variables achieved statistical significance, Table 3. Thus, the data-driven multivariable regression analysis could not be performed.

**Table 3:** Predicting successful tapering at 18 months in the tapering group

Possible baseline predictors	Univariate analysis RR (95% CI)	p-value	Clinically-driven analysis RR (95% CI)	p-value
Female	1.10 (0.59–2.03)	0.772		
Age (years)	1.01 (0.99–1.03)	0.478		
Body mass index (kg/m^2^)	1.01 (0.94–1.08)	0.855	1.03 (0.96–1.10)	0.423
Diagnosis				
RA	1 (ref.)	–		
PsA	0.94 (0.38–2.36)	0.902		
AxSpA	1.23 (0.62–2.43)	0.555		
Disease duration (months)	0.99 (0.95–1.03)	0.610		
On csDMARD	0.60 (0.30–1.17)	0.133	0.54 (0.28–1.04)	0.067
On MTX	0.62 (0.32–1.22)	0.170		
On ≥ 2 csDMARD	1.59 (0.38–6.65)	0.523		
Duration of baseline biologic (months)	1.00 (0.99–1.00)	0.285		
Previous attempt–taper TNFi	0.65 (0.30–1.42)	0.281		
CRP (mg/L)	0.97 (0.85–1.10)	0.635		
In remission^a^	1.44 (0.51–4.12)	0.492		
TNFi drug-level category				
High	0.94 (0.48–1.83)	0.845	0.96 (0.49–1.87)	0.909
Intermediate	1 (ref.)	–	1 (ref.)	–
Low	0.56 (0.24–1.33)	0.190	0.51 (0.22–1.18)	0.114

*RR* relative risk, *95% CI* 95% confidence interval, *p* p-value, *kg* kilogram, *m*^*2*^ square meter, *RA* rheumatoid arthritis, *PsA* psoriatic arthritis, *axSpA* axial spondyloarthritis, *csDMARD* conventional synthetic disease-modifying anti-rheumatic drugs, *MTX* methotrexate, *TNFi* tumour necrosis factor inhibitor, *CRP* C-reactive protein, *mg* milligram, *L* liter

^a^Evaluated as Disease Activity Score (DAS)28-CRP < 2.6 for RA and PsA, and Ankylosing Spondylitis Disease Activity Score (ASDAS) < 1.3 for axSpA

The clinically-driven multivariable regression model included the pre-selected variables: BMI, on csDMARD, and TNFi drug-level category. Treelet transformation was not necessary as no correlation was demonstrated. The clinically-driven prediction analysis did not identify any statistically significant predictors as presented in Table 3. Area under the receiver operator curve was 0.65 (95% CI 0.52–0.77), Supplementary figure S1.

## Discussion

To our knowledge, this study is the first to assess TNFi drug-levels and ADAb during disease activity-guided TNFi tapering compared to TNFi continuation in patients with IA. The study demonstrated a decrease in TNFi drug-levels in the tapering group between baseline and month 18; thus, indicating acceptable compliance to the tapering algorithm. Disease activity was equivalent between groups at 18 months despite the difference in TNFi drug-levels. Moreover, the frequency of ADAb during the TNFi tapering process was extremely low.

Strengths of these analyses are: based on data from a large randomised, controlled trial with a study population resembling the real-life outpatient population, limited patients lost to follow-up, and available blood samples for the majority of patients throughout the study period.

It was expected, that TNFi drug-levels would decrease during tapering based on data from previous trials [17, 25]. L’Ami et al. evaluated adalimumab tapering (from every second week to every third week) to adalimumab standard dose in 78 patients with RA with high adalimumab drug-levels (> 8 µg/mL) at baseline [25]. A significantly lower adalimumab drug-level at 28 weeks was demonstrated in the tapering group; between group difference: 2.6 µg/mL (95% CI 1.2–4.1). Similar to our results, disease activity was not deteriorated in the tapering group despite a lower adalimumab drug-level. A prospective observational study by Chen et al. found lower adalimumab drug-levels at 24 weeks (5.5 mg/mL vs 2.6 mg/mL, respectively) after dose-halving in 64 patients with RA [17]. However, loss of LDA was observed in 24% (15/64) of patients at 24 weeks. Nonetheless, in the majority of the existing literature tapering has been proven effective in reducing TNFi dose while maintaining acceptable disease activity [1–10] which is reassuring for patients and physicians.

A noteworthy finding in our study is the very low frequency of ADAb which only were present in patients treated with adalimumab or infliximab. This is in line with existing literature, as the highest ADAb rate have been reported for adalimumab and infliximab [26]. To our knowledge, ADAb development during a tapering process have only been assessed by Chen et al.: 5% (3/64) had adalimumab ADAb at 24 weeks compared to none at baseline [17]. Other tapering studies have reported ADAb at baseline ranging from: adalimumab ADAb 0–10% [23, 27, 28], infliximab 16% [23], whereas no etanercept ADAb have been reported in line with our findings [23, 28]. Differences in ADAb frequency between studies could be due to variations across assay methods [26]. Moreover, blood samples were only taken as trough samples by Chen et al.; therefore, ADAb could theoretically be underestimated in the remaining studies (including our study). Another notable finding in our study was that only one out of three patients with ADAb at baseline not could taper their TNFi; the remaining two patients flared but managed to regain LDA at an escalated, but still tapered, TNFi dose. A higher frequency of patients with axSpA (14%) developed ADAb in our study compared to RA (6%) and PsA (6%). One could speculate if the higher frequency of ADAb in axSpA could be due to less concomitant csDMARD treatment in this patient group as concomitant csDMARD treatment have been demonstrated to decrease the risk of ADAb development [29]. Nonetheless, the tapering process in our study did not result in an increased frequency of ADAb development nor in loss of therapeutic response. These data can be used to qualify the discussion on tapering between physicians and patients.

In our study, successful tapering was achieved by 32% of patients in the tapering group at 18 months. A prediction analysis did not find baseline TNFi drug-level category to predict successful tapering. In line with our results, combined data from the DRESS-RA trial and an observational cohort study did not demonstrate any predictive value of baseline adalimumab, etanercept or infliximab drug-levels for achieving successful tapering or discontinuation in patients with RA [23]. The STRASS trial reported no predictive value of baseline adalimumab or etanercept drug-levels when assessing flare risk in patients with RA who underwent TNFi tapering or continuation [28]. PREDICTRA evaluated adalimumab tapering to adalimumab withdrawal in patients with RA and found no association between baseline adalimumab drug-level and flare risk at week 40 in either of the trial groups [27]. Nor did the POET study, who evaluated adalimumab withdrawal, demonstrate a predictive value of baseline adalimumab trough-level or baseline ADAb when assessing the risk of flare at one year in patients with RA [30]. In light of these data, TNFi drug-level and/or presence of ADAb cannot be used to guide the decision on who to taper. However, future research with larger trials is needed to explore the topic further.

An important limitation to the study is that only patients treated with a TNFi at baseline could be included as assays for measuring abatacept and tocilizumab drug-levels not were available. Another limitation is that TNFi drug-levels not could be analysed as a continuous variable due to truncation of low and high values; therefore, the variable was categorised into three groups based on existing literature [15, 23, 24] or the manufactures recommendation. However, categorisation increases the risk of information loss [21, 22] which can lead to overlooking important differences e.g., is very high TNFi drug-levels at baseline a potential predictor for achieving successful tapering at 18 months? A large study population decreases the risk of overlooking important differences but increases the risk of finding differences where none exist. As these analyses were based on data from 129 patients, the potential risk of overlooking important differences due to categorisation of the variable TNFi drug-levels is judged to be less relevant than the violation of the linear model assumptions with truncated continuous data.

Another aspect to consider is that blood sampling was not performed as trough levels. A sensitivity analysis was performed to assess the potential implication of the time span between the last biological dose and blood sampling. Reassuringly, the results did not alter the conclusions.

Lastly, the baseline frequency of ADAb was very low in our study as could be expected in a study population in sustained LDA and on stable arthritis treatment. One could question if this ‘well-treated’ population would be less likely to develop ADAb despite TNFi tapering; thereby, introducing a degree of selection bias? Future research with larger trials is therefore needed to explore if ADAb have any clinically relevant implications during the tapering process.

## Conclusion

In conclusion, a shift in TNFi drug-level categories was observed between baseline and month 18 in the tapering group resulting in more patients with low levels and fewer with high levels. Despite the difference in TNFi drug-levels at 18 months, disease activity remained equivalent. Moreover, the frequency of ADAb during the TNFi tapering process was extremely low. Our data does not support using TNFi drug-level category and/or presence of ADAb to guide the tapering decision but future research with larger trials is needed to explore the topic further.

## Supplementary Information

Below is the link to the electronic supplementary material.

Supplementary file1 (PDF 4798 KB)

## Data Availability

Anonymised data will be shared upon reasonable request.
