# Supplementary material for: Exploring TNFi drug-levels and anti-drug antibodies during tapering among patients with inflammatory arthritis: secondary analyses from the randomised BIODOPT trial
Source: Rheumatol Int. 2024 Jul 24;44(10):1897–908. doi: 10.1007/s00296-024-05665-7 (PMC11392959; doi:10.1007/s00296-024-05665-7)
Supplement: Supplementary file 1 — Supplementary file1 (PDF 4798 KB) [file 296_2024_5665_MOESM1_ESM.pdf]

SUPPLEMENTARY APPENDIX

Exploring TNFi drug-levels and anti-drug antibodies during tapering among patients with inflammatory arthritis:

Secondary analyses from the randomised BIODOPT trial

CONTENT

Supplementary Table S1……………..…….……………..………………………………………………………………..S3
Supplementary Table S2…………………………..………………………………………………………………..S4
Supplementary Table S3………………………..…………………………………………………………………..S5
Supplementary Data S1………………….………….....………………………………………………………………..S6
Supplementary Table S4……………………………………………………………………..……………………..S7
Supplementary Table S5……………………………………………………..………………………………….. …S8
Supplementary Table S6……………………………………………………..………………………………….....S9
Supplementary Figure S1…...……………………………………………..…………………………………....S10
Supplementary Data S2………………………...………………………………………………………………….S11

Supplementary Table S1: Pre-defined TNFi drug-level categories used in these analyses

Drug	Low	Intermediate	High
Adalimumab (1)	<5 mg/L	5.0-8.0 mg/L	>8 mg/L
Certolizumab pegol (2)	<14.7 mg/L[1]	14.7-40.0 mg/L	>40.0 mg/L
Etanercept (1)	<1.8 mg/L	1.8-4.6 mg/L	>4.6 mg/L
Golimumab (3)	<1.0 mg/L	1.0-3.0 mg/L	>3 mg/L
Infliximab (1)	<1.66 mg/L[1]	1.66-5.0 mg/L	>5.0 mg/L

[1]: Lowest detectable level in accordance with the manufacture.

References

1. Bouman C, van Herwaarden N, van den Hoogen F, van der Maas A, van den Bemt B, den Broeder AA. Prediction of successful dose reduction or discontinuation of adalimumab, etanercept, or infliximab in rheumatoid arthritis patients using serum drug-levels and antidrug antibody measurement. Expert Opin Drug Metab Toxicol. 2017;13:597–603.
2. Jani M, Isaacs JD, Morgan AW, Wilson AG, Plant D, Hyrich KL, et al. High frequency of antidrug antibodies and association of random drug-levels with efficacy in certolizumab pegol-treated patients with rheumatoid arthritis: Results from the BRAGGSS cohort. Ann Rheum Dis. 2017;76:208–13.
3. Pedersen L, Szecsi PB, Johansen PB, Bjerrum PJ. Evaluation of therapeutic drug monitoring in the clinical management of patients with rheumatic diseases: Data from a retrospective single-center cohort study. Biol Targets Ther. 2020;14:115–25.

Supplementary Table S2: Sensitivity analyses on primary and secondary outcomes at 18 months.

Variable	Tapering group, *N=88*	Control group, *N=41*	Between group difference
	N (%)	***N (%)***	***RR (95%CI)***
TNFi drug-level category:			
High[a]	19 (22%)	17 (42%)	0.53 (0.31 to 0.90)
Intermediate[b]	15 (17%)	6 (15%)	1.18 (0.49 to 2.82)
Low[c]*, n (%)*	54 (61%)	18 (44%)	1.37 (0.94 to 2.01)
Presence of ADAb	14 (16%)	4 (10%)	1.47 (0.52 to 4.21)

N: number, 95%CI: 95% confidence interval, TNFi: tumour-necrosis factor inhibitor, ADAb: anti-drug antibodies.

[a]: Analysed as 'high' or 'not high' i.e., 'not high' equals intermediate AND low TNFi drug-levels.

[b]: Analysed as 'intermediate' or 'not intermediate' i.e., 'not intermediate' equals low AND high TNFi drug-levels.

[c]: Analysed as 'low' or 'not low' i.e., 'not low' equals intermediate AND high TNFi drug-levels.

Supplementary Table S3: Changes in drug-levels, and anti-drug antibodies from baseline to 18 months.

**Variable**	**Tapering group, *N=88***			**Control group, *N=41***		
	Baseline	***18 months***	***Change***	***Baseline***	***18 months***	***Change***
	N (%)	***N (%)***	***Proportional difference (95%CI)***	***N (%)***	***N (%)***	***Proportional difference (95%CI)***
TNFi drug-level category:						
High[a]	31 (35%)	19 (22%)	-14% (-27% to -1%)	16 (39%)	17 (41%)	2% (-20% to 25%)
Intermediate[b]	29 (33%)	25 (28%)	-5% (-19% to 10%)	15 (37%)	10 (24%)	-12% (-36% to 12%)
Low[c]	28 (32%)	44 (50%)	18% (5% to 31%)	10 (24%)	14 (34%)	10% (-12% to 31%)
Presence of ADAb	3 (4%)	4 (5%)	1% (-5% to 1%)	0 (0%)	0 (0%)	0% (-2% to 2%)

N: number, 95%CI: 95% confidence interval, TNFi: tumour-necrosis factor inhibitor, ADAb: anti-drug antibodies.

Change is analysed as 18 month – baseline.

[a]: Analysed as 'high' or 'not high' i.e., 'not high' equals intermediate AND low TNFi drug-levels.

[b]: Analysed as 'intermediate' or 'not intermediate' i.e., 'not intermediate' equals low AND high TNFi drug-levels.

[c]: Analysed as 'low' or 'not low' i.e., 'not low' equals intermediate AND high TNFi drug-levels.

Supplementary Data S1: Additional results

TNFi drug-levels at 12 months

At 12 months, similar results were demonstrated as significantly fewer patients in the tapering group had high TNFi drug-levels compared to the control group, RR 0.37 (95%CI: 0.21 to 0.66), ***Supplementary Table S4***. No significant difference in intermediate or low TNFi drug-level was observed between groups at 12 months. The sensitivity analysis still found a significant difference in high TNFi drug-levels between groups at 12 months; moreover, a significant difference in low TNFi drug-levels was observed (RR: 1.60 [95%CI: 1.06 to 2.43]) which most likely is due to the handling of missing data i.e., missing values were imputed as low TNFi drug-level in the sensitivity analyses, ***Supplementary Table S5***. When looking at changes in TNFi drug-levels between baseline and month 12, a significant shift was observed in the tapering group resulting in fewer patients with high drug-levels (proportional difference: -19% [95%CI: -31% to -7%]) and low drug-levels (proportional difference: 19% [95%CI: 7% to 32%]), ***Supplementary Table S6***.

Supplementary Table S4: Drug-levels, and anti-drug antibodies at 12 months.

Variable	Tapering group, *N=88*	Control group, *N=41*	Between group difference
	N (%)	*N (%)*	*RR (95%CI)*
TNFi drug-level category:			
High[a]	14 (16%)	18 (44%)	0.37 (0.21 to 0.66)
Intermediate[b]	29 (33%)	9 (22%)	1.45 (0.78 to 2.69)
Low[c]	45 (51%)	14 (34%)	1.51 (0.97 to 2.35)
Presence of ADAb	4 (5%)	0 (0%)	-

N: number, 95%CI: 95% confidence interval, TNFi: tumour-necrosis factor inhibitor, ADAb: anti-drug antibodies.

[a]: Analysed as ‘high’ or ‘not high’ i.e., ‘not high’ equals intermediate AND low TNFi drug-levels.

[b]: Analysed as ‘intermediate’ or ‘not intermediate’ i.e., ‘not intermediate’ equals low AND high TNFi drug-levels.

[c]: Analysed as ‘low’ or ‘not low’ i.e., ‘not low’ equals intermediate AND high TNFi drug-levels.

Supplementary Table S5: Sensitivity analyses on primary and secondary outcomes at 12 months.

Variable	Tapering group, *N=88*	Control group, *N=41*	Between group difference
	N (%)	***N (%)***	***RR (95%CI)***
TNFi drug-level category:			
High[a]	14 (16%)	18 (44%)	0.37 (0.21 to 0.66)
Intermediate[b]	18 (20%)	7 (17%)	1.21 (0.54 to 2.70)
Low[c]	56 (64%)	16 (39%)	1.60 (1.06 to 2.43)
Presence of ADAb	14 (16%)	2 (5%)	2.95 (0.70 to 12.44)

N: number, 95%CI: 95% confidence interval, TNFi: tumour-necrosis factor inhibitor, ADAb: anti-drug antibodies.

[a]: Analysed as 'high' or 'not high' i.e., 'not high' equals intermediate AND low TNFi drug-levels.

[b]: Analysed as 'intermediate' or 'not intermediate' i.e., 'not intermediate' equals low AND high TNFi drug-levels.

[c]: Analysed as 'low' or 'not low' i.e., 'not low' equals intermediate AND high TNFi drug-levels.

Supplementary Table S6: Changes in drug-levels, and anti-drug antibodies from baseline to12 months.

Variable	Tapering group, *N=88*			Control group, *N=41*		
	Baseline ***N (%)***	***18 months*** ***N (%)***	***Change*** ***Proportional difference (95%CI)***	***Baseline*** ***N (%)***	***18 months*** ***N (%)***	***Change*** ***Proportions (95%CI)***
TNFi drug-level category:						
High[a]	31 (35%)	14 (16%)	-19% (-31% to -7%)	16 (39%)	18 (44%)	5% (-13% to 22%)
Intermediate[b]	29 (33%)	29 (33%)	0% (-15% to 15%)	15 (37%)	9 (22%)	-15% (-37% to 8%)
Low[c]	28 (32%)	45 (51%)	19% (7% to 32%)	10 (24%)	14 (34%)	10% (-9% to 28%)
Presence of ADAb	3 (4%)	4 (5%)	1% (-5% to 7%)	0 (0%)	0 (0%)	0% (-2% to 2%)

N: number, 95%CI: 95% confidence interval, TNFi: tumour-necrosis factor inhibitor, ADAb: anti-drug antibodies.
Change is analysed as 12 month – baseline.
[a]: Analysed as ‘high’ or ‘not high’ i.e., ‘not high’ equals intermediate AND low TNFi drug-levels.
[b]: Analysed as ‘intermediate’ or ‘not intermediate’ i.e., ‘not intermediate’ equals low AND high TNFi drug-levels.
[c]: Analysed as ‘low’ or ‘not low’ i.e., ‘not low’ equals intermediate AND high TNFi drug-lev

Supplementary figure S1: Area under the receiver operator curve with 95% confidence interval for the clinical-driven multivariable prediction model.

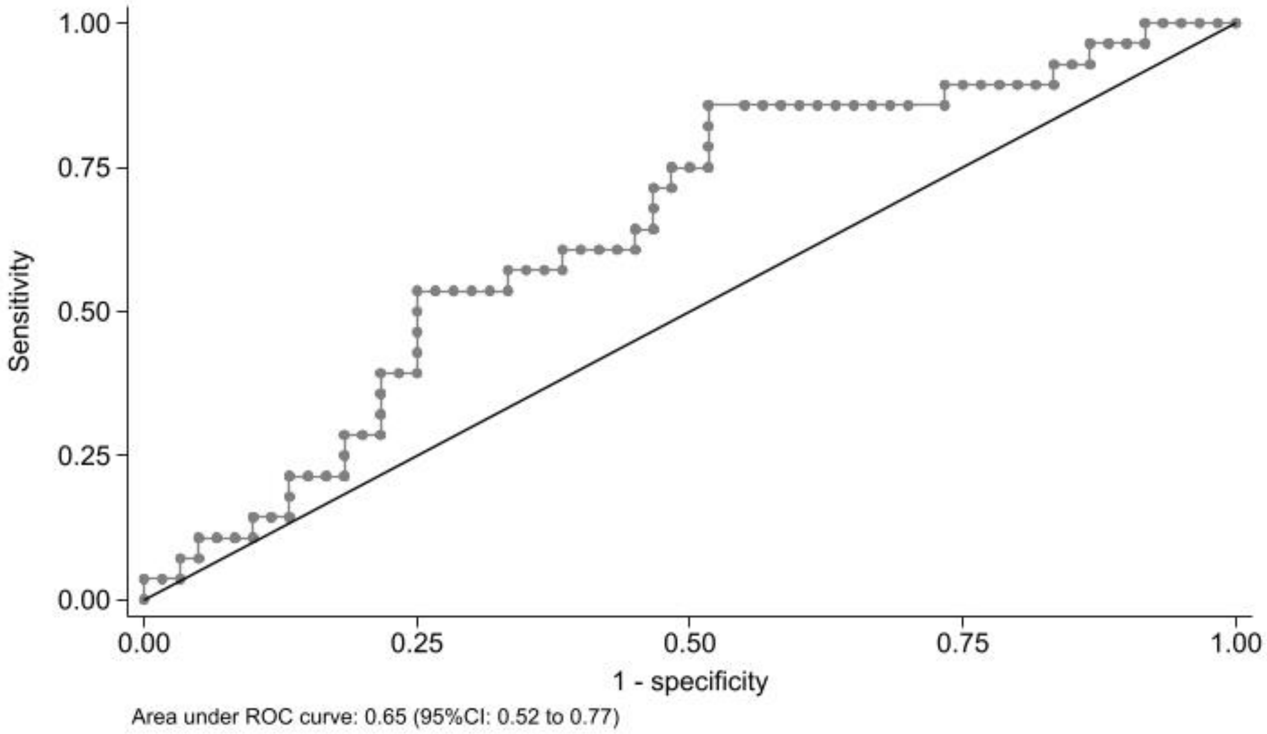

Supplementary Data S2: Statistical Analysis Plan for Secondary Analyses based on the BIODOPT Trial: Exploring tumour necrosis factor inhibitor drug levels during disease activity-guided tapering among patients with inflammatory arthritis - Secondary analyses on blood samples from the randomised BIODOPT trial

Statistical Analysis Plan for Secondary Analyses based on the BIODOPT Trial:

Exploring tumour necrosis factor inhibitor drug levels during disease activity-guided tapering among patients with inflammatory arthritis:

Secondary analyses on blood samples from the randomised BIODOPT trial

Line Uhrenholt[1,2,3], Karen Buch Lauridsen[4], Kirsten Duch[1,5], Lene Dreyer[1,2], Robin Christensen[3,6], Ellen-Margrethe Hauge[7,8], Anne Gitte Loft[7,8], Mads NB Rasch[7], Hans Christian Horn[9], Peter C Taylor[10], Kaspar R Nielsen[4], Salome Kristensen[1,2]

AFFILIATIONS

[1]: Department of Rheumatology, Aalborg University Hospital, Aalborg, Denmark

[2]: Department of Clinical Medicine Aalborg University, Aalborg, Denmark

[3]: Section for Biostatistics and Evidence-Based Research, the Parker Institute, Bispebjerg and Frederiksberg Hospital, Copenhagen, Denmark

[4]: Department of Clinical Immunology, Aalborg University Hospital, Aalborg, Denmark

[5]: Unit of Epidemiology and Biostatistics, Aalborg University Hospital, Aalborg, Denmark

[6]: Research Unit of Rheumatology, Department of Clinical Research, University of Southern Denmark, Odense University Hospital, Odense, Denmark

[7]: Department of Rheumatology, Aarhus University Hospital, Aarhus, Denmark

[8]: Department of Clinical Medicine, Aarhus University, Aarhus, Denmark

[9]: Department of Rheumatology, Odense University Hospital, Odense, Denmark

[10]: Nuffield Department of Orthopaedics, Rheumatology and Musculoskeletal Sciences, University of Oxford, Oxford, UK

Section 1: Administrative Information

Title and trial registration

Statistical analysis plan (SAP) for the secondary analyses from the randomised BIODOPT trial entitled "Exploring drug levels of tumour necrosis factor inhibitor trajectory levels during disease activity-guided tapering among patients with inflammatory arthritis".

Trial registration: EudraCT number: 2017-001970-41.

SAP version

Version 1.1

October 25th, 2022

Previously, a detailed description of the statistical analysis plan of the BIODOPT 18 months data have been finalised (version 1.0, September 14th, 2021).

Protocol version

This document has been written based on information contained in the study protocol version 10, dated September 5th, 2019.

SAP revisions

None.

Roles and responsibility

LU and RC designed and wrote this SAP in accordance to the SPIRIT/CONSORT and SAP statement for randomised trials (1–3). LU and RC are responsible for analysis of the trial results according to the SAP.

Signatures

Line Uhrenholt 1/11 - 22

MD, PhD Student, Line Uhrenholt Date

01.11.22

MSc, Biostatistician, Kirsten Duch Date

2022, Nov 03

MSc, PhD, Senior Biostatistician, Professor, Robin Christensen Date

Section 2: Study Methods

Background and rationale

BIODOPT was designed as a pragmatic, multicentre, randomised controlled, open-label, parallel-group trial, assessing the impact of tapering biologicals in patients with inflammatory arthritis ([IA] i.e., rheumatoid arthritis [RA], psoriatic arthritis [PsA], and axial spondyloarthritis [axSpA]) in sustaining remission or low disease activity (LDA). Traditionally, if tolerated, standard doses of biologics are maintained lifelong after sustained remission/LDA are reached; however, recent studies indicate that a significant proportion can successfully taper biologic dose while maintaining a stable low disease activity. However, little is known about changes in drug levels, anti-drug antibodies (ADAb) and cytokine levels during disease activity-guided tapering of biologics in patients with IA.

Trial design

BIODOPT was designed as a pragmatic, multicentre, randomised controlled, open-label, parallel-group trial of 18 months duration. Eligible patients were diagnosed with RA, PsA or axSpA treated with bDMARDs while maintaining remission/LDA during the past 12 months. Patients were randomised in a ratio of 2:1 to either disease activity-guided tapering of biologics (tapering group) or continuation of biologics as usual care (continuation group).

Methods

At each trial visit (i.e. baseline, 4 month, 8 month, 12 month, 18 month and additional visits due to possible flare), biological material (blood) was collected in connection to routine blood samples. Thus, the timing of blood sampling was not chosen to be at a fixed interval with respect to the timing of last biologic dose administration. However, the date for the last dose of biologics before the blood sample was noted at each trial visit in the dedicated electronic case rapport form (e-CRF) in REDCap. The biological material (blood) was stored in the Danish Rheumatology Biobank (Dansk Reuma Biobank) with the purpose of exploratory analysis. All analyses on the biological material (blood) will be performed after the 18-months follow-up visit is completed for all participants. Drug concentration and anti-drug antibodies will be measured by IDKmonitor enzyme-linked immunoassorbant assays, Immundiagnostik AG, Bensheim, Germany. Cytokines will be measured by MILLIPLEX Multiplex assays, Merck KGaA, Darmstadt, Germany.

TNFi drug levels

The table below gives an overview of previously defined TNFi trough levels.

Drug	Low	Intermediate	High
Adalimumab (4)	<5 mg/L	5.0-8.0 mg/L	>8 mg/L
Certolizumab pegol (5)	<14.7 mg/L[1]	14.7-40.0 mg/L	>40.0 mg/L
Etanercept (4)	<1.8 mg/L	1.8-4.6 mg/L	>4.6 mg/L
Golimumab (6)	<1.0 mg/L	1.0-3.0 mg/L	>3 mg/L
Infliximab (4)	<1.66 mg/L[1]	1.66-5.0 mg/L	>5.0 mg/L

[1]: Lower detectable level in accordance with the manufacture.

Anti-drug antibodies

Based on previous research, anti-drug antibodies were considered to be positive when values were ≥10 arbitrary units/ml according to the manufacturer's recommendation.

Cytokines

By expert opinion, the following cytokines were judged to be of particular interest:

- TNF-alpha (TNF-α)
- Soluble TNF-α-1-receptor (sTNF-1R)
- Soluble TNF-α-2-receptor (sTNF-2R)
- IL-6
- Soluble IL-6-receptor (sIL6-R)
- IL17A
- IL23

Successful tapering definition

A patient is considered to have successfully tapered biologic dose if the following points are fulfilled:

- Did not deviate from the trial protocol
- The subject's biologic dose is reduced by ≥50% compared to baseline
- The subject remains in LDA at 18 months:
    - RA and PsA: Disease Activity Score28-C-Reactive Protein (DAS28-CRP) ≤3.2
    - AxSpA: Ankylosing Spondylitis Disease Activity Score (ASDAS) <2.1

Baseline characteristics included in the regression analyses

- Allocated intervention:
    - Tapering group
- Baseline demographics:
    - Female sex
    - Age (continuous)
    - Body Mass Index (kg/m^2) (continuous)
- Inflammatory arthritis characteristics:
    - Diagnosis: RA, PsA, or axSpA
    - Disease duration (years)
    - On ≥2 csDMARDs
    - On MTX
    - Repeated bDMARD failure i.e. currently on biological agent number ≥3
    - Duration of baseline biological therapy (continuous)
    - Previous attempt to taper bDMARD
    - C-reactive protein (CRP) (continuous)
    - In remission (RA and PsA: DAS28-CRP < 2.6 or axSpA: ASDAS < 1.3)
- Laboratory assessments:
    - TNFi drug level category:
        - High
        - Intermediate
        - Low
    - Presence of ADAb
    - Cytokines (continuous):
        - TNF-α
        - sTNF-1R
        - sTNF-2R
        - IL-6
        - sIL6-R
        - IL-17A
        - IL-23

Aims

The aim of this BIODOPT spin-off study is to explore changes in TNFi drug levels, presence of ADAb, and cytokine levels in patients with IA in sustained LDA/remission who taper their biological treatment compared to continuation of biologics as usual care.

Objectives

The primary objective will be to compare the tapering group, relative to the continuation group, on TNFi drug level categories at 18 months.

The key secondary objective will be to compare the tapering group, relative to the continuation group, on the number of patients having presence of ADAb at 18 months.

Other secondary objectives will be to compare the groups according to the following cytokine levels at 18 months: TNF-α, sTNF-1R, sTNF-2R, IL-6, sIL6-R, IL-17A, and IL-23.

Tertiary outcomes will be to compare the tapering group, relative to the continuation group, on TNFi drug level categories, presence of ADAb, and cytokine levels at 12 months.

Primary outcome

TNFi drug level categories at 18 months in the BIODOPT trial.

Key secondary outcomes

The number of patients having presence of ADAb at18 months.

Other secondary outcomes

Cytokines levels at 18 months for the following:

- TNF-α
- sTNF-1R
- sTNF-2R
- IL-6
- sIL6-R
- IL-17A
- IL-23

Tertiary outcomes

TNFi drug level categories, presence of ADAb, and cytokine levels at 12 months.

Exploratory outcomes

Exploratory analyses will be performed in an attempt to identify possible baseline predictors for successful biologic tapering including the baseline characteristics listed above.

Timing of outcome assessments

Analyses will be performed on blood samples from baseline, 12 months, and 18 months visit.

Timing of final analysis

Final analysis will be performed collectively and published in one paper (expected submission date October 2022).

Section 3: Analysis

Analysis populations

The analyses will be based on the Intention to Treat (ITT) population. The ITT principle asserts the effect of a treatment policy (that is, the planned treatment regimen), rather than the actual treatment given (i.e., it is independent of treatment adherence). Accordingly, participants allocated to a treatment group (X_{Taper} and X_{Control}, respectively) should be followed up, assessed, and analysed as members of that group, irrespective of their adherence to the planned course of treatment (i.e., independent of withdrawals and cross-over phenomena).

Baseline patient characteristics

Baseline characteristics will be presented as illustrated in **Outline Table 1**. Categorical variables will be summarised with count and percentages and continuous variables will be summarised with mean and standard deviation (SD) if approximately normally distributed; otherwise with median and interquartile range (IQR). Formal statistical tests will not be performed; however, apparent imbalances will be noted and, if necessary, evaluated based on standardised differences rather than *P*-values.

Analysis methods

All analyses will be performed in accordance with this pre-specified SAP (3,7) and reported in accordance with the recommendations of the EQUATOR network (8); i.e. the appropriate CONSORT statement (1,2) and TRIPOD recommendation (9).

Continuous outcomes (i.e., cytokine levels) will be analysed using linear mixed models for repeated measures. Group (tapering *vs* continuation), diagnosis (RA, PsA, *or* axSpA), biologic failure history (<3 previous biologics, *or* ≥3 previous biologics), centre status (Aalborg, Aarhus, Odense, *or* Silkeborg), and time point (0, 12, and 18 months) as well as the interaction between group and time will be included as fixed effects, and id as random intercept. Moreover, the baseline value of the relevant variable (e.g., cytokine level) will be included as a covariate to reduce the random variation. Contrasts between groups will be presented as least squares means with 95% confidence interval (95%CI). Missing outcome variables will be handled indirectly in the repeated-measures linear mixed model.

The categorical outcome TNFi drug level category will be analysed as binomial i.e., 1) Low drug level versus intermediate/high drug level, 2) intermediate drug level versus low/high drug level, and 3) High drug level versus intermediate/low drug level. Mixed Poisson regression with robust variance estimator will be applied for analyses of the binomial outcomes (i.e., TNFi drug level categories and ADAb). Fixed effects in the model include: group (tapering *vs* continuation), diagnosis (RA, PsA, *or*

axSpA), biologic failure history (<3 previous biologics, *or* ≥3 previous biologics), centre status (Aalborg, Aarhus, Odense, *or* Silkeborg), and time point (0, 12, and 18 months) as well as the interaction between group and time, and id as random intercept. Moreover, the baseline value of the relevant variable (e.g., TNFi drug level category) will be included as a covariate to reduce the random variation. Contrasts between groups will be presented as relative risk (RR) and 95%CI. Missing data will be handled in the primary analysis by "single-step" non-responder imputation; thus, patients with missing outcome variables for ADAb will be imputed as *not having developed TNFi ADAb* and patients with missing data for TNFi drug level category will be imputed as *intermediate TNFi drug level* as this is the 'normal range' for most patients.

For the prediction analyses, missing values for successful tapering will be analysed as trial failure i.e. the patient *did not achieve successful tapering at 18 months*. The pre-specified baseline characteristics of interest (potential predictors) will be analysed using univariable modified Poisson regression with robust variance estimator (10) and presented as relative risk (RR), 95%CI and p-value. Continuous predictors will be grouped to identify relevant nonlinear predictors. If nonlinear relation is identified, predictors will be categorized in clinically relevant groups based on expert opinion and treated as a categorical variable. Non-grouped continuous predictors will be included as linear predictors. Multivariable regression analyses with modified Poisson regression using a robust variance estimator will be performed in two ways; first with a clinical-driven approach including the following baseline characteristics (selected by expert opinion): tapering group, BMI, TNFi drug level categories, presence of ADAb, and on concomitant csDMARDs at baseline. Second, a data-driven approach including all variables with a univariate p-value <0.10 will be performed. Results from the multivariable regression analyses will be presented by RR with 95%CI. Pairwise correlation between predictors will be investigated and if relevant, treelet transformation will be used to further investigate the correlation structure (11). Leave-one-out cross validation will be performed and assessed using the concordance index (c-index) for the binary outcome identical to the area under the receiving operator characteristic curve (AUC) (12). All analyses will be performed using commercially available statistical software SAS version 9.4 or STATA, version 16).

Sensitivity analyses

Sensitivity analyses on the primary and secondary outcomes will be performed to evaluate potential implications of missing data. For continuous outcomes (i.e. cytokine levels), missing data will be handled as baseline observation carried forward. Missing data for ADAb will be imputed as *having developed ADAb* and missing data for TNFi drug level category will be imputed as *low TNFi drug level*. Moreover, a sensitivity analysis on the primary outcome will be performed to explore potential

implications of blood sampling time (measured continuous [in days]) in relation to the last dose of biologics before the blood sample was performed. If nonlinear relation is identified, the variable 'blood sampling time' will be categorised in clinically relevant groups and treated as a categorical variable.

References

1. Moher D, Hopewell S, Schulz KF et al. CONSORT 2010 explanation and elaboration: Updated guidelines for reporting parallel group randomised trials. BMJ. 2010;340:c869.
2. Piaggio G, Elbourne DR, Altman DG et al. Reporting of noninferiority and equivalence randomized trials: an extension of the CONSORT statement. JAMA. 2012;308:2594–604.
3. Gamble C, Krishan A, Stocken D, Lewis S, Juszczak E, Doré C, et al. Guidelines for the content of statistical analysis plans in clinical trials. JAMA. 2017;318:2337–43.
4. Bouman C, van Herwaarden N, van den Hoogen F, van der Maas A, van den Bemt B, den Broeder AA. Prediction of successful dose reduction or discontinuation of adalimumab, etanercept, or infliximab in rheumatoid arthritis patients using serum drug levels and antidrug antibody measurement. Expert Opin Drug Metab Toxicol. 2017;13:597–603.
5. Jani M, Isaacs JD, Morgan AW, Wilson AG, Plant D, Hyrich KL, et al. High frequency of antidrug antibodies and association of random drug levels with efficacy in certolizumab pegol-treated patients with rheumatoid arthritis: Results from the BRAGGSS cohort. Ann Rheum Dis. 2017;76:208–13.
6. Pedersen L, Szecsi PB, Johansen PB, Bjerrum PJ. Evaluation of therapeutic drug monitoring in the clinical management of patients with rheumatic diseases: Data from a retrospective single-center cohort study. Biol Targets Ther. 2020;14:115–25.
7. Christensen R, Langberg H. Statistical principles for prospective study protocols: design, analysis, and reporting. Int J Sport Phys Ther. 2012;7:504–11.
8. Christensen R, Bliddal H, Henriksen M. Enhancing the reporting and transparency of rheumatology research: a guide to reporting guidelines. Arthritis Res Ther. 2013;15:109.
9. Collins GS, Reitsma JB, Altman DG, Moons KGM. Transparent reporting of a multivariable prediction model for individual prognosis or diagnosis (TRIPOD): The TRIPOD statement. BMJ [Internet]. 2014;350:g7594. Available from: http://dx.doi.org/doi:10.1136/bmj.g7594
10. Zou G. A Modified Poisson Regression Approach to Prospective Studies with Binary Data. Am J Epidemiol. 2004;159:702–6.
11. Gorst-rasmussen A. tt: Treelet transform with Stata. Dep Math Sci Aalborg Univ. 2011;Research R.
12. Shipe ME, Deppen SA, Farjah F, Grogan EL. Developing prediction models for clinical use using logistic regression: An overview. J Thorac Dis. 2019;11:S574–84.

Section 4: Manuscript outline

The manuscript outline will include the following documents:

- Outline Figure 1: Flow-diagram over the study period.
- Outline Table 1: Baseline demographics, disease characteristics and laboratory assessments in the ITT population treated with a tumour necrosis factor inhibitor at baseline.
- Outline Figure 2: Mock-up (simulated data visualisation) of TNFi drug level categories at baseline and 18 months analysed on the ITT population.
- Outline Table 2: TNFi drug level categories, antidrug antibodies and cytokines at 18 months analysed based on the ITT population.
- Outline Table 3: Univariable and multivariable regression analyses for prediction of successful tapering at 18 months follow-up.
- Outline Appendix Table 1: Sensitivity analyses on the primary and secondary outcomes at 18 months analysed based on the ITT population.
- Outline Appendix Table 2: TNFi drug level categories, antidrug antibodies and cytokines at 12 months analysed based on the ITT population.

Outline Figure 1: Flow-diagram over the study period.

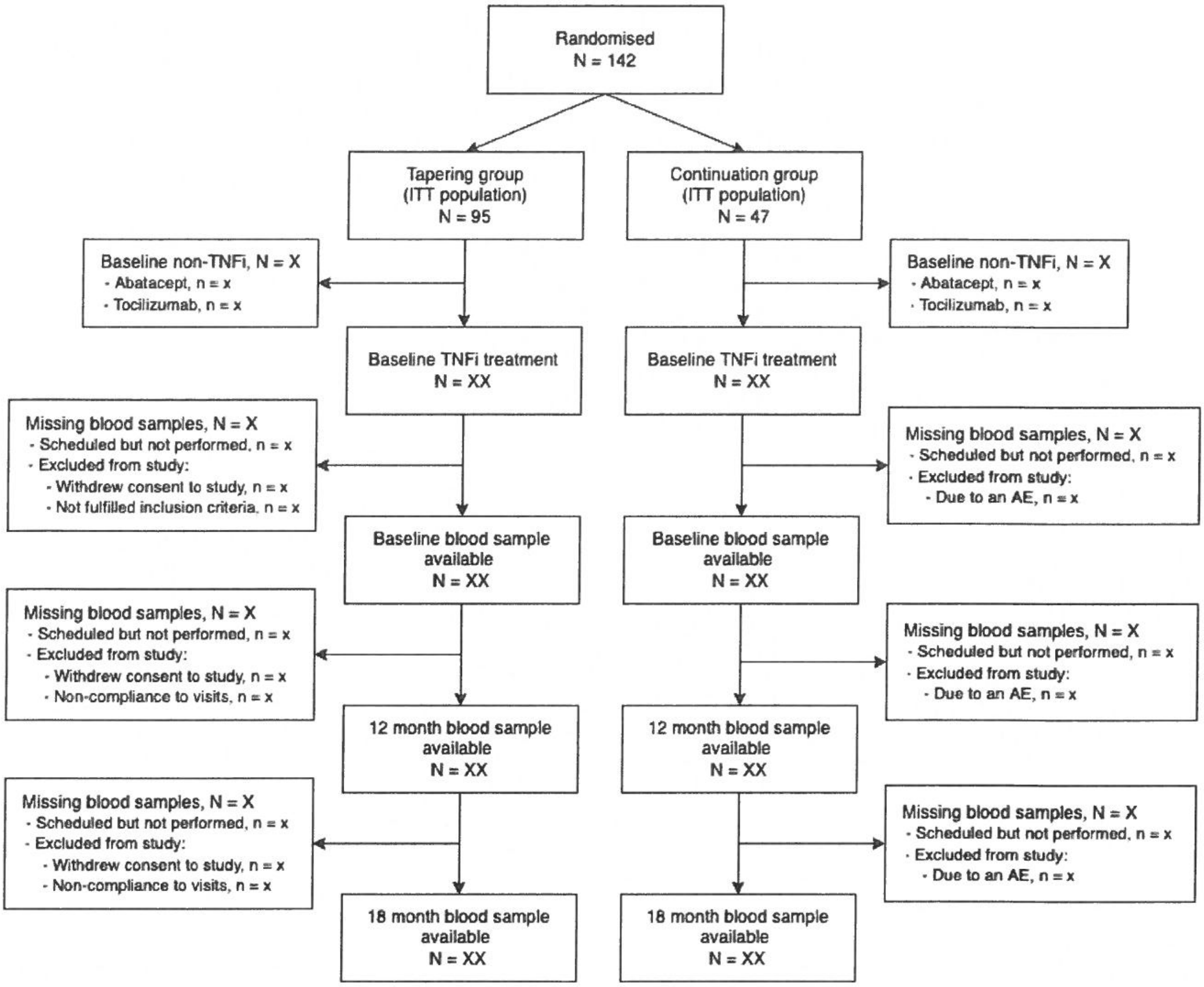

Outline Table 1: Baseline demographics, disease characteristics and laboratory assessments in the ITT population treated with a tumour necrosis factor inhibitor at baseline.

Variable	Tapering group (*N*=?)	Continuation group (*N*=?)
General characteristics		
Female, n (%)		
Age (years)		
Body Mass Index (kg/m^2)		
Arthritis characteristics		
Diagnosis:		
RA, n (%)		
PsA, n (%)		
axSpA, n (%)		
Disease duration (years)		
On ≥2 csDMARDs, n (%)		
On MTX, n (%)		
Repeated biologics failure[1], n (%)		
Duration of baseline biologic (years)		
Previous attempt to taper TNFi, n (%)		
CRP (mg/L)		
In remission[2], n (%)		
Laboratory assessments		
Baseline TNFi drug level category:		
High, n (%)		
Intermediate, n (%)		
Low, n (%)		
Presence of ADAb at baseline, n (%)		
Baseline cytokines:		
TNF-α, pg/mL		
sTNF-1R, pg/mL		
sTNF-2R, pg/mL		
IL-6, pg/mL		
sIL6-R, pg/mL		
IL17A, pg/mL		
IL-23, pg/mL		

Values are means and standard deviations (SD) unless otherwise stated. N: number, kg: kilogram, m^2: square metre, RA: rheumatoid arthritis, PsA: psoriatic arthritis, axSpA: axial spondyloarthritis, csDMARD: conventional synthetic disease-modifying anti-rheumatic drugs, MTX: methotrexate, TNFi: tumour necrosis factor inhibitor, CRP: C-reactive protein, mg: milligram, L:litre, ADAb: anti-drug antibodies, TNF-α: tumour necrosis factor alpha, sTNF-1R: soluble tumour necrosis factor 1 receptor, sTNF-2R: soluble tumour necrosis factor 2 receptor, IL: interleukin, sIL6-R: soluble interleukin 6 receptor.

[1]: Patients on biological agent number ≥3.

[2]: Evaluated as Disease Activity Score (DAS)28-CRP <2.6 for RA and PsA and Ankylosing Spondylitis Disease Activity Score (ASDAS) <1.3 for axSpA.

Outline Figure 2: Mock-up (simulated data visualisation) of TNFi drug level categories at baseline and 18 months analysed on the ITT population.

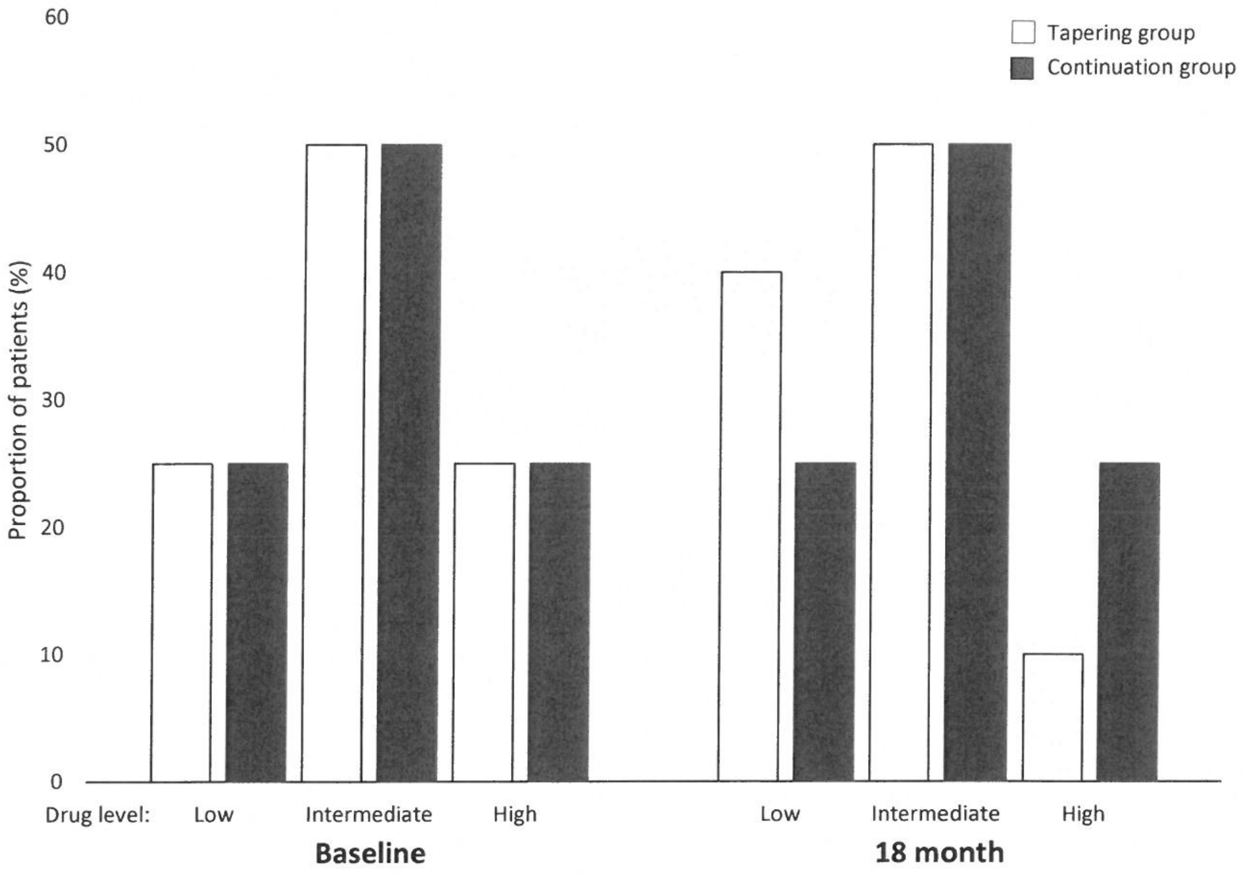

Outline Table 2: TNFi drug level categories, antidrug antibodies and cytokines at 18 months analysed based on the ITT population.

**Variable**	**18 months**		
	**Tapering group** *N=?*	**Continuation group** *N=?*	**Between group difference** **RR (95%CI)**
TNFi drug level category:			
High, n (%)			
Intermediate, n (%)			
Low, n (%)			
Presence of ADAb, n (%)			
Cytokines:			
TNF-α, LSMeans (SE)			
sTNF-1R, LSMeans (SE)			
sTNF-2R, LSMeans (SE)			
IL-6, LSMeans (SE)			
sIL6-R LSMeans (SE)			
IL-17A, LSMeans (SE)			
IL-23, LSMeans (SE)			

N: number, RR: relative risk, 95%CI: 95% confidence interval, TNFi: tumour-necrosis factor inhibitor, ADAb: anti-drug antibodies, TNFα: tumour-necrosis factor alpha, LSMeans: least squares means, SE: standard error, sTNF-1R: soluble tumour-necrosis factor 1 receptor, sTNF-2R: soluble tumour-necrosis factor 2 receptor, IL: interleukin, sIL6-R: soluble interleukin 6 receptor.

Outline Table 3: Univariable and multivariable regression analyses for prediction of successful tapering at 18 months follow-up.

Possible baseline predictors	Univariate analysis RR (95%CI)	p	Multivariable analysis Data-driven RR (95%CI)	Multivariable analysis Clinical-driven RR (95%CI)
Tapering group				
Female				
Age (years)				
Body Mass Index (kg/m^2)				
Diagnosis:				
RA				
PsA				
axSpA				
Disease duration (years)				
On csDMARD				
On MTX				
Repeated biologics failure				
Duration of baseline biologic (years)				
Previous attempt to taper TNFi				
CRP (mg/L)				
In remission[1]				
TNFi drug level category:				
High				
Intermediate				
Low				
Presence of ADAb				
Cytokines:				
TNF-α (pg/mL)				
sTNF-1R (pg/mL)				
sTNF-2R (pg/mL)				
IL-6 (pg/mL)				
sIL6-R (pg/mL)				
IL-17A (pg/mL)				
IL-23 (pg/mL)				

RR: relative risk, 95%CI: 95% confidence interval, p: p-value, kg: kilogram, m^2: square metre, RA: rheumatoid arthritis, PsA: psoriatic arthritis, axSpA: axial spondyloarthritis, csDMARD: conventional synthetic disease-modifying anti-rheumatic drugs, MTX: methotrexate, TNFi: tumour necrosis factor inhibitor, CRP: C-reactive protein, mg: milligram, L: litre, ADAb: anti-drug antibodies, TNFα: tumour-necrosis factor alpha, sTNF-1R: soluble tumour-necrosis factor 1 receptor, sTNF-2R: soluble tumour-necrosis factor 2 receptor, IL: interleukin, IL6-R: interleukin 6 receptor.

[1]: Evaluated as Disease Activity Score (DAS)28-CRP <2.6 for RA and PsA and Ankylosing Spondylitis Disease Activity Score (ASDAS) <1.3 for axSpA.

Outline Appendix Table 1: Sensitivity analyses on the primary and secondary outcomes at 18 months analysed based on the ITT population.

**Variable**	**18 months**		
	**Tapering group** *N=?*	**Continuation group** *N=?*	**Between group difference RR (95%CI)**
TNFi drug level category:			
High, n (%)			
Intermediate, n (%)			
Low, n (%)			
Presence of ADAb, n (%)			
Cytokines:			
TNF-α, LSMeans (SE)			
sTNF-1R, LSMeans (SE)			
sTNF-2R, LSMeans (SE)			
IL-6, LSMeans (SE)			
sIL6-R LSMeans (SE)			
IL-17A, LSMeans (SE)			
IL-23, LSMeans (SE)			

N: number, RR: relative risk, 95%CI: 95% confidence interval, TNFi: tumour-necrosis factor inhibitor, ADAb: anti-drug antibodies, TNFα: tumour-necrosis factor alpha, LSMeans: least squares means, SE: standard error, sTNF-1R: soluble tumour-necrosis factor 1 receptor, sTNF-2R: soluble tumour-necrosis factor 2 receptor, IL: interleukin, sIL6-R: soluble interleukin 6 receptor.

Outline Appendix Table 2: TNFi drug level categories, antidrug antibodies and cytokines at 12 months analysed based on the ITT population.

Variable	12 months		
	**Tapering group** *N=?*	**Continuation group** *N=?*	**Between group difference RR (95%CI)**
TNFi drug level category:			
High, n (%)			
Intermediate, n (%)			
Low, n (%)			
Presence of ADAb, n (%)			
Cytokines:			
TNF-α, LSMeans (SE)			
sTNF-1R, LSMeans (SE)			
sTNF-2R, LSMeans (SE)			
IL-6, LSMeans (SE)			
sIL6-R LSMeans (SE)			
IL-17A, LSMeans (SE)			
IL-23, LSMeans (SE)			

N: number, RR: relative risk, 95%CI: 95% confidence interval, TNFi: tumour-necrosis factor inhibitor, ADAb: anti-drug antibodies, TNFα: tumour-necrosis factor alpha, LSMeans: least squares means, SE: standard error, sTNF-1R: soluble tumour-necrosis factor 1 receptor, sTNF-2R: soluble tumour-necrosis factor 2 receptor, IL: interleukin, sIL6-R: soluble interleukin 6 receptor.
